# Role of different nutrient profiling models in identifying targeted foods for front-of-package food labelling in Brazil

**DOI:** 10.1017/S1368980019005056

**Published:** 2021-04

**Authors:** Ana Clara Duran, Camila Zancheta Ricardo, Laís Amaral Mais, Ana Paula Bortoletto Martins

**Affiliations:** 1 Center for Food Studies and Research (NEPA), University of Campinas, Campinas, SP 13083-852, Brazil; 2 Center for Epidemiological Studies in Nutrition and Health (NUPENS), University of Sao Paulo, Sao Paulo 01246-904, Brazil; 3 Brazilian Institute for Consumer Defense (IDEC), Sao Paulo, SP 05002-050, Brazil

**Keywords:** Nutrient profiling, Labelling, Processed foods, Food supply, Packaged foods

## Abstract

**Objective:**

To compare the degree of strictness and agreement of different nutrient profiling models (NPM) used to identify which foods would be required to show front-of-package (FOP) warning labels.

**Design:**

Using data of 11 434 packaged foods found in the five largest food retailers in Brazil, we used two published NPM: the Pan American Health Organization (PAHO) model and the NPM used in the Chilean nutritional FOP labelling policy, and compared them with a NPM proposed by the Brazilian National Health Surveillance Agency (Anvisa). The proportion of foods that would be required to show FOP warning labels was calculated overall and by food category. We also tested whether a modified version of the PAHO NPM would behave similarly to the original version.

**Setting:**

Brazil.

**Results:**

Two-thirds of the packaged products (62 %) would receive FOP warning labels under the PAHO NPM, as compared with 45 % of products using the proposed Anvisa NPM and 41 % if the Chilean NPM was applied. The PAHO NPM identified more foods high in critical nutrients such as sweetened dairy and non-dairy beverages, canned vegetables and convenience foods. Overall agreement between models was considered good with kappa coefficient ranging from 0·57 to 0·92 but was lower for some food categories.

**Conclusions:**

We found variations in the degree of strictness and agreement between assessed NPM. The PAHO NPM identified more foods and beverages high in sugar which are among the top contributors to sugar and energy intake in Brazil.

In response to the rising consumption of unhealthy foods such as ultra-processed foods^(1,2)^ and the link with weight gain^(3)^, public health scholars and advocates have promoted public policies aiming to improve food environments by reducing the availability and affordability of unhealthy foods^(4)^. Regulatory and fiscal policies such as increasing the price of unhealthy foods, restricting food marketing and improving access to information in packaged foods at the point of purchase through clear, simple nutritional labelling have been recognised as useful tools to encourage healthier food choices^(5,6)^.

For instance, recently, Chile implemented the inclusion of front-of-package (FOP) warning labels as a tool to help consumers make healthier food choices^(7)^. Other countries such as Mexico, Israel and Canada are following the Chilean lead^(8)^. Regulatory efforts are also currently being discussed in Brazil aiming to both review the overall nutritional labelling in the country and include FOP warning labels in packaged foods and beverages. Such efforts come along with other measures to help push for a price increase of unhealthy foods and beverages as implemented elsewhere^(9,10)^.

Warning labels, as compared with other types of available FOP labelling, can more efficiently help consumers choose between healthier and less healthy food options^(6,11)^. A nutrient profiling model (NPM) that is able to adequately distinguish between healthy and unhealthy foods needs to accompany food and nutrition regulatory policies, particularly related to correctly identifying foods with excessive amounts of critical nutrients such as sugar, fats and sodium, in order for such policies to be effective^(5,6)^. According to the WHO, ‘nutrient profiling is the science of classifying or ranking foods according to their nutritional composition for reasons related to preventing disease and promoting health’^(12)^. Overall, NPM can help promote public health dietary goals ensuring consistency with national guidelines to promote a healthy diet and fight non-communicable diseases and obesity^(12,13)^. Characteristics of NPM include the eligibility criteria (e.g. will all foods and beverages be eligible to be classified or just certain groups?), which nutrients are considered, the definition of food categories, the selected reference amounts, the established thresholds, etc^(14,15)^. A NPM should also be objective, transparent and easy to implement and enforce to be used in various regulatory food and nutrition policies^(12,13)^.

Regional criteria for NPM aimed at identifying foods high in nutrients associated with non-communicable diseases have been developed with the help of experts in nutrition in different WHO regions, including the Pan American Health Organization (PAHO)^(16)^. For the FOP nutritional labelling regulation, Chile authorities developed their own NPM based on the food consumption distribution of the Chilean population^(7)^.

Considering the currently regulatory scenario in Brazil, we aimed to compare the degree of strictness and agreement of different NPM to determine which NPM could identify unhealthy foods that would be eligible to show a FOP warning label in Brazil.

## Methods

### Database of Brazilian packaged foods

This is a cross-sectional study that used data from a sample of packaged foods found in the five largest Brazilian chain supermarkets. Supermarkets were selected as the source of the data collection because they account for a large share (59 %) of the energy consumed by Brazilians^(17)^. The five food retail chains with the greatest market share found in Brazil were identified using annual sales data in food retail organised by *Euromonitor International* in 2016^(18)^. The top five retailers in Brazil account for 69·7 % of the edible grocery banner sales in the country^(19)^. São Paulo, located in the Southeast region of the country, was chosen as the primary study area because it is the largest city in Brazil^(20)^. As one of the food retail chains only had stores in the Northeast region of the country, data collection at this chain was conducted in Salvador, their largest market and largest city in the Northeast region^(20)^.

### Stores selection

Data on the location of every store of these five retail chains in Brazil in the cities of São Paulo and Salvador were gathered from each company’s website and the addresses were geocoded. The neighbourhood of each store was defined as one-km buffer (using Euclidean distance) around the store location. We used information on income from the household top earner from the 2010 Brazilian Census^(20)^. Stores were then stratified by tertiles of neighbourhood income, and the largest store of each retail chain in the first and third tertiles were selected to ensure socio-economic representativeness in the sample, except for one chain that only allowed data collection in its distribution centre, where all products sold in the chain were available. Formal permission was obtained from all the supermarkets chains included in the current study.

Data were collected between April and July 2017 by trained fieldworkers, according to methods proposed by Kanter *et al.* ^(21)^. All packaged foods and beverages were included, and around 13 000 different items had all sides of their package photographed. Data were entered between July and November 2017 by trained nutritionists in an online platform using a template developed by researchers from the Instituto de Nutrición y Tecnología de los Alimentos, Chile, and the University of North Carolina at Chapel Hill, United States of America, adapted to be used in Brazil^(21)^. Information on product name, brand, flavour, company address, package size, nutrition facts panel information, list of ingredients and information about reconstitution (when applied) was entered. For 10 % of the sample, data were double entered by the same person and repeated by a second person for intra- and inter-rater reliability analyses, respectively.

Products entered during the training phase and with duplicate records were excluded, resulting in 12 956 products. For the current analysis, products available in more than one package size (*n* 358), multipack with different items (*n* 86), products without nutrition facts panel (*n* 815), products without the list of ingredients (*n* 178) and products with missing values for portion size and/or energies (*n* 85) were also excluded (online Supplementary file 1).

For beverages and dessert instant mixes, concentrated juices, powdered milks, tea, coffee and instant soups, we reconstituted the volume of the product to its as-consumed form to get the nutrition information in 100 g or ml. Assessed packaged foods were then categorised into food groups considering previous studies^(21)^ and the Brazilian food supply context (online Supplementary file 2).

### Nutrient profiling models

We compared three NPM: a NPM proposed by PAHO^(16)^, a NPM proposed by the National Health Surveillance Agency (*Agência Nacional de Vigilância Sanitária* – Anvisa) as part of the current effort to update the nutritional labelling legislation in Brazil^(22)^ and the NPM proposed by Chilean authorities that has been used in their FOP nutritional labelling regulation^(7)^ and considered elsewhere^(23)^. The effort led by Anvisa in Brazil aims to improve nutrition labelling in Brazil and include an easier way to understand FOP nutritional labelling system. Anvisa, among other duties, is responsible for regulating food labelling in the country. Nutritional labelling is mandatory in Brazilian packaged foods and beverages and requires that foods and beverages sold in Brazil have a nutrition facts panel with information per serving and the content of the following nutrients: energies, carbohydrate, total fats, saturated fats, trans fats, total fibre and sodium. If the food or beverage presents claims for other nutrients, manufacturers should also disclose the amount of these nutrients in the product^(24)^.

We chose to use the PAHO NPM for policy coherence as it better aligns with the Brazilian Food-Based Dietary Guidelines than other published NPM^(25)^. It was published in 2016 to be used in food and nutrition policies, including FOP warning labels, in Latin America. This NPM considers the level and degree of industrial processing, according to the NOVA classification^(26)^, as an eligibility criteria. Only processed and ultra-processed foods are eligible to be classified as containing or not excessive amounts of five nutrients: free sugars, total fats, saturated fats, trans fat and sodium. In addition, the presence of non-nutritive sweeteners (NNS) in the list of ingredients is also considered in the model. This NPM considers nutrient thresholds to determine whether a product has a high content of each nutrient by using the ratio between the content of the critical nutrient and the content of energy in the product and follows cut-offs for nutrient content ratios that the WHO has set to prevent obesity and chronic diseases^(16)^. For the PAHO NPM, we identified which products contained added sugars and NNS using a search based on keywords in the list of ingredients of each food or beverage.

Anvisa has proposed its own NPM to identify unhealthy foods that should receive FOP labels, and we tested it against the PAHO NPM^(22)^. The thresholds in this model were based in a projection of the Brazilian population nutrition requirements in 2020 and on WHO recommendations to prevent obesity and chronic diseases^(16)^. Anvisa’s NPM evaluates the content of free sugar, sodium and saturated fats in 100 g or 100 ml of foods and beverages, respectively. In this model, sugars, salts, vinegars, herbs, coffees, frozen fruits and vegetables, frozen and chilled meats, baby foods and foods for special dietary uses are not eligible to be regulated and receive FOP warning labels. In response to the known link between some non-communicable diseases such as cardiovascular diseases, infertility, endometriosis, gallstones, Alzheimer’s disease, diabetes and some types of cancer^(27)^ and the consumption *trans*-fatty acids (TFA), the WHO has called for the elimination of TFA from the global food supply. Thus, Anvisa, as part of the discussion to update and improve nutrition labelling regulation in Brazil, has proposed to ban TFA from the Brazilian food supply as opposed to improving TFA labelling.

Finally, we tested and compared the PAHO NPM and the NPM proposed by Anvisa with the NPM proposed by Chilean authorities to identify which foods would receive a FOP warning label in that country^(7)^. Chile has been a leader in the implementation of FOP warning labels and has been followed by other countries such as Canada, Israel, Mexico, Peru and Uruguay^(8)^. In addition, their proposed NPM has been adopted in Peru^(23)^ and has heavily influenced the nutritional labelling discussions in Brazil, which drove us to test whether such model would be adequate to classify the Brazilian packaged food supply. In the Chilean NPM, only foods and beverages with added sodium, sugar or saturated fat were eligible to receive FOP warning labels for ‘high in’ critical nutrients (energies, total sugars, total fats, saturated fats and sodium) (Law 20.606/2015)^(7)^. Culinary ingredients, as sugar, salt, oils, butter and milk creams, were only included if the product had the addition of another critical nutrient in excessive amounts (for instance, butter made with milk cream and salt is eligible to be regulated and receives a warning label for high content of sodium if this nutrient is in excess – however, it does not receive a warning label related to the high content of fats).

For the Chilean and the PAHO NPM, in order to determine whether a food was eligible to receive a warning label, we ran a search based on keywords in the list of ingredients of each food or beverage. Briefly, keywords for added sugar included sugar, honey, syrups, molasses, maltodextrin, glucose, fructose, and concentrated fruit and vegetables juices and also included sweets like chocolates and milk sweet. Keywords for salt included salt, sodium chloride, cheeses and processed meats. Keywords for fat included oils, olives, butter, creams, and animal and vegetal fats. Keywords for NNS included aspartame, saccharin, sucralose, cyclamate, acesulfame k, stevia, polydextrose, maltitol, mannitol, isomaltose, neotame, xylitol, thaumatin and advantame. All searches were made in Portuguese.

Considering the specific needs of the proposed labelling regulatory process that need a NPM that identifies which foods should or not receive FOP warning labels, we also tested whether a modified version of the PAHO NPM that uses a previously tested eligibility criteria would behave similarly to the PAHO NPM. In this modification, a food or beverage was eligible to be regulated and therefore receive FOP warning labels based on the Chilean nutritional labelling law eligibility criteria (Law 20.606/2015)^(7)^. For this modified PAHO NPM, we used the same targeted five nutrients (free sugar, total fats, saturated fats, trans fat, and sodium) and applied the same threshold levels as the PAHO NPM. For the modified-PAHO NPM, in addition to added sugar and NNS, we searched for foods that contained added salt and fat using the same keywords we employed for the search to determine eligibility to be labelled in the Chilean NPM.

Table 1 shows the characteristics and cut-offs of the NPM we considered in the current study.

**Table 1:** Characteristics and cut-off points of the assessed nutrient profiling models used to identify unhealthy foods in the food supply

Nutrients	PAHO	Anvisa	Chilean	Modified-PAHO
Total fat	≥30 % of total energy from fat	–	–	≥30 % of total energy from fat
Saturated fat	≥10 % of total energy from saturated fat	≥4 g/100 g; ≥2 g/100 ml	≥4 g/100 g; ≥3 g/100 ml	≥10 % of total energy from saturated fat
Trans fat	≥1 % of total energy from trans fat	–	–	≥1 % of total energy from trans fat
Sugar	≥10 % of total energy from free sugars	≥10 g/100 g; ≥5 g/100 ml (free sugars)	≥10 g/100 g; ≥5 g/100 ml (total sugar)	≥10 % of total energy from free sugars
Sodium	≥4·2 kJ (1 kcal)	≥400 mg/100 g; ≥200 mg/100 ml	≥400 mg/100 g; ≥100 mg/100 ml	≥1 mg/4·2 kJ (1 kcal)
Energies	–	–	≥1150 kJ (275 kcal); ≥233 kJ (70 kcal)	–
Non-nutritive sweeteners	Presence	–	–	Presence
Eligibility for being rated	Processed and ultra-processed foods and beverages, according to NOVA classification	All foods and beverages. Exceptions: fruits and vegetables, meats, salt, herbs, oil, sugar, vinegar, coffee, baby foods, meal replacement shakes, diet products or with other nutrient restriction	Foods and beverages with added sugar, Na or fat	Foods and beverages with added sugar, Na or fat

PAHO, Pan American Health Organization; modified-PAHO, modified Pan American Health Organization nutrient profiling model; Anvisa, National Health Surveillance Agency (Agência Nacional de Vigilância Sanitária).

### Reliability analyses

Intra- and inter-rater reliability were calculated in 10 % of the sample using the intra-class coefficient and were found to be excellent (intra-class coefficient ≥ 0·90 for all assessed nutrients)^(28)^.

### Comparison of the nutrient profiling models

We determined the degree of strictness of each NPM by the number and proportion (percentage and 95 % CI) of foods and beverages that had a high content of the critical nutrients assessed in each model and, therefore, that would be considered unhealthy and receive FOP warning labels. Analyses were done overall and by food groups. We determined the agreement between the various models using cross-classification analysis (i.e. number and proportion of food products classified similarly or differently between any two models) and the Cohen’s kappa statistics. Agreement was interpreted as follows: 0·01–0·20 – slight; 0·21–0·40 – fair; 0·41–0·60 – moderate; 0·61–0·80 – substantial; and 0·81–1·00 – almost perfect or perfect agreement^(28)^.

Although the PAHO NPM considers free sugars, this information is not available on food labels of products sold in Brazil. We thus estimated the amount of free sugars using the method proposed by PAHO that considers the information on the amount of total sugars declared on food labels^(16)^. In this method, foods are classified by the information available on the nutrition facts panel (total sugars or added sugars) and by food category. For instance, if information on total sugars is available and the product has no or a minimal amount of naturally occurring sugars, such as soda and sports drinks, then the total amount of added sugars is considered free sugars. For milk or yogurt with any type of sugar in the list of ingredients, 50 % of the declared added sugars were considered free sugars, so lactose, galactose and other types of naturally occurring sugars were not considered free sugars. However, because the information for total sugars in food labels is not mandatory in Brazil, analyses that considered total or free sugars were conducted for a sub-sample of products that provided such information.

We could not compare products according to a high content of TFA and the presence of NNS across different NPM due to their absence in two of the assessed NPM. We thus described the presence of NNS in Brazilian packaged foods overall and by food categories. Data on the presence of TFA in the Brazilian food supply are available elsewhere^(29)^.

## Results

The proportion of foods in each food category is presented in Table 2, as well as the proportion of products in each category that contain a high content in at least one of the critical nutrients, according to different NPM. Two-thirds of the packaged Brazilian food supply would be eligible to receive FOP warning labels for any critical nutrient if the PAHO NPM was adopted (62·2 %; 95 % CI 61·3, 63·1) as compared with 45·1 % of the foods if the NPM proposed by Anvisa was adopted (95 % CI 44·2, 46·0) and 41·7 % (95 % CI 40·8, 42·6) of the assessed foods in Brazil considering the Chilean NPM. Sweetened beverages, sweetened dairy beverages, canned vegetables and convenience foods were the food groups with the largest differences in terms of identified foods high in critical nutrients when we compared the various NPM. Using the PAHO NPM, we were able to identify more beverages high in sugar and those with the presence of NNS as well as convenience foods and canned vegetables high in sodium than with the other tested NPM.

**Table 2:** Proportion of foods with a high content of at least one of the critical nutrients according to different nutrient profiling models, overall and by food category, Brazil, 2017*

Food category	Full sample		PAHO		Anvisa		Chilean	
	*n*	%	%	95 % CI	%	95 % CI	%	95 % CI
Non-dairy beverages								
Carbonated beverages	106	0·9	57·5	47·9, 66·6	24·5	17·2, 33·6	24·5	17·2, 33·6
Fruit juices	150	1·3	8·7	5·1, 14·4	12·0	7·7, 18·3	10·7	6·6, 16·7
Fruit-flavoured drinks	220	1·9	73·6	67·4, 79·0	2·3	0·9, 5·4	2·3	0·9, 5·4
Nectars	160	1·4	28·8	22·3, 36·3	5·0	2·5, 9·7	10·6	6·7, 16·4
Coffee and tea	94	0·8	5·3	2·2, 12·2	0·0	–	0·0	–
Other beverages	286	2·5	75·9	70·6, 80·5	15·7	12·0, 20·4	12·9	9·5, 17·4
Dairy								
Sweetened dairy beverages	483	4·2	82·2	78·5, 85·4	6·2	4·4, 8·7	16·1	13·1, 19·7
Unsweetened dairy beverages	181	1·6	14·9	10·4, 20·9	16·0	11·4, 22·1	0·0	–
Cheese and cheese spreads	607	5·3	99·5	98·5, 99·8	94·6	92·4, 96·1	32·9	29·3, 36·8
Salty snacks	356	3·1	94·7	91·8, 96·6	91·6	88·2, 94·0	95·2	92·4, 97·0
Sweets and desserts								
Cookies	747	6·5	93·7	91·7, 95·2	82·5	79·6, 85·0	98·9	97·9, 99·5
Candies and desserts	1220	10·7	79·8	77·5, 82·0	50·2	47·4, 53·0	67·4	64·7, 70·0
Fruit preserves	414	3·6	23·9	20·0, 28·3	13·0	10·1, 16·6	34·1	29·6, 38·8
Convenience or ready-to-eat foods								
Frozen/fresh ready-to-eat foods	795	7·0	92·6	90·5, 94·2	68·1	64·7, 71·2	54·8	51·4, 58·3
Processed meats	810	7·1	96·7	95·2, 97·7	87·7	85·2, 89·7	60·2	56·8, 63·6
Sauces and dressings	801	7·0	83·0	80·3, 85·5	72·7	69·5, 75·6	60·0	56·6, 63·4
Bakery products	595	5·2	85·5	82·5, 88·2	57·3	53·3, 61·2	77·8	74·3, 81·0
Breakfast cereals and granola bars	308	2·7	64·9	59·4, 70·1	49·0	43·5, 54·6	93·8	90·5, 96·0
Culinary ingredients								
Sugar and non-nutritive sweeteners	106	0·9	42·5	33·4, 52·1	0·0	–	14·2	8·7, 22·2
Oils and fats	351	3·1	27·9	23·5, 32·9	31·9	27·2, 37·0	12·8	9·7, 16·7
Other minimally processed and processed foods								
Canned vegetables	345	3·0	89·6	85·9, 92·4	60·6	55·3, 65·6	15·9	12·4, 20·2
Cereals, beans, other grain products	735	6·4	12·1	9·9, 14·7	13·9	11·6, 16·6	6·1	4·6, 8·1
Meat, poultry, seafood and egg	577	5·0	0·0	–	0·0	–	0·0	–
Nuts and seeds	80	0·7	47·5	36·8, 58·5	77·5	67·0, 85·4	35·0	25·3, 46·1
Packaged fruits and vegetables	907	7·9	0·0	–	0·0	–	0·0	–
Total	11 434	100·0	62·2	61·3, 63·1	45·1	44·2, 46·0	41·7	40·8, 42·6

PAHO, Pan American Health Organization; Anvisa, National Health Surveillance Agency (Agência Nacional de Vigilância Sanitária); CI, confidence interval.

* Information for the content of free or total sugars was considered in 10 % of the sample. For the PAHO and Anvisa nutrient profiling models (NPM), free sugars were calculated from the information on total sugars available on products. For the Chilean NPM, the information on total sugars content available on food products nutrition facts panels was considered. It is not possible to calculate the kappa coefficient when there is no observation in one of the categories.

Tables 3–5 show the degree of strictness of each model for sugars, sodium and saturated fats, respectively, and the agreement between the three assessed NPM. We were able to determine the agreement between the assessed models for these nutrients only as these are the ones considered in the Anvisa and Chile NPM. We found lower agreement between the PAHO NPM and the Anvisa or the Chilean NPM for high content of sugars (the overall kappa statistics was 0·62 and 0·67, respectively). For sodium, a fair to poor agreement was found when the Chilean and the PAHO models (*κ* = 0·42) were compared; and for saturated fat, a poor agreement (*κ* = 0·57) was found for the comparison between the PAHO and the Chilean NPM as compared with the other two comparisons (overall kappa coefficients ranged from 0·72 to 0·75).

**Table 3:** Agreement between classifications made by assessed nutrient profiling models for high content of sugars applied to Brazilian packaged foods*, 2017

Food category	*N*	High content of sugars* (%)			PAHO/Anvisa			PAHO/Chilean			Anvisa/Chilean		
		PAHO	Anvisa	Chilean	% AG	*κ*	95 % CI	% AG	*κ*	95 % CI	% AG	*κ*	95 % CI
Non-dairy beverages													
Carbonated beverages	51	58·8	51·0	51·0	92·2	0·84	0·70, 0·99	92·2	0·84	0·70, 0·99	100·0	1·00	
Fruit juices	59	15·3	25·4	25·4	89·8	0·69	0·47, 0·91	89·8	0·69	0·47, 0·91	100·0	1·00	
Fruit-flavoured drinks	83	62·7	6·0	6·0	43·4	0·07	0·01, 0·14	43·4	0·07	0·01, 0·14	100·0	1·00	
Nectars	22	77·3	36·4	36·4	59·1	0·29	0·04, 0·54	59·1	0·29	0·04, 0·54	100·0	1·00	
Coffee and tea	5	0·0	0·0	0·0									
Other beverages	140	50·7	17·9	18·6	64·3	0·29	0·17, 0·41	65·0	0·31	0·19, 0·43	99·3	0·98	0·93, 1·000
Dairy													
Sweetened dairy beverages	64	78·1	3·1	40·6	25·0	0·02	–0·01, 0·04	62·5	0·32	0·16, 0·48	62·5	0·09	–0·03, 0·21
Unsweetened dairy beverages	14	0·0	0·0	0·0									
Cheese and cheese spreads	1	0·0	0·0	0·0									
Salty snacks	57	1·8	1·8	1·8	100·0	1·0		100·0	1·00		100·0	1·00	
Sweets and desserts													
Cookies	126	65·9	66·7	67·5	97·6	0·95	0·89, 1·00	98·4	0·96	0·92, 1·00	99·2	0·98	0·95, 1·00
Candies and desserts	296	70·6	54·1	58·8	83·5	0·66	0·58, 0·74	84·8	0·67	0·59, 0·76	95·3	0·90	0·86, 0·95
Fruit preserves	31	45·2	29·0	32·3	83·9	0·66	0·41, 0·92	87·1	0·73	0·50, 0·97	96·8	0·92	0·78, 1·00
Convenience or ready-to-eat foods													
Frozen/fresh ready-to-eat foods	30	3·3	0·0	0·0	96·7	0·0		96·7	0·0		.	.	
Processed meats	3	0·0	0·0	0·0									
Sauces and dressings	76	47·4	35·5	35·5	85·5	0·71	0·55, 0·86	85·5	0·71	0·55, 0·86	100·0	1·00	
Bakery products	33	18·2	3·0	15·2	84·9	0·25	–0·15, 0·65	97·0	0·89	0·68, 1·00	87·9	0·30	–0·16, 0·76
Breakfast cereals and granola bars	90	71·1	72·2	72·2	98·9	0·97	0·92, 1·00	98·9	0·97	0·92, 1·00	100·0	1·00	
Culinary ingredients													
Sugar and non-nutritive sweeteners	2	0·0	0·0	0·0									
Oils and fats	5	0·0	0·0	0·0									
Other minimally processed and processed foods													
Canned vegetables	9	0·0	0·0	0·0									
Cereals, beans, other grain products	22	0·0	0·0	0·0									
Meat, poultry, seafood and egg	0	0·0	0·0	0·0									
Nuts and seeds	0	0·0	0·0	0·0									
Packaged fruits and vegetables	23	0·0	0·0	0·0									
Total	1242	51·8	34·5	38·1	80·8	0·62	0·58, 0·66	83·6	0·67	0·64, 0·71	96·4	0·92	0·90, 0·94

PAHO, Pan American Health Organization; Anvisa, National Health Surveillance Agency (Agência Nacional de Vigilância Sanitária); AG, agreement; CI, confidence interval.

* Information for the content of free or total sugars was considered in 10 % of the sample. For the PAHO and Anvisa nutrient profiling model (NPM), free sugars were calculated from the information on total sugars available on products. For the Chilean NPM, the information on total sugars content available on food products nutrition facts panels was considered. It is not possible to calculate the kappa coefficient when there is no observation in one of the categories.

**Table 4:** Agreement between classifications made by assessed nutrient profile models for high content of sodium applied to Brazilian packaged foods, 2017*

Food category	*N*	High content of sodium (%)			PAHO/Anvisa			PAHO/Chilean			Anvisa/Chilean		
		PAHO	Anvisa	Chilean	% AG	*κ*	95 % CI	% AG	*κ*	95 % CI	% AG	*κ*	95 % CI
Non-dairy beverages													
Carbonated beverages	105	24·8	0·0	0·0	75·2	0·00		75·2	0·00		.	.	
Fruit juices	149	2·7	2·0	0·7	99·3	0·85	0·57, 1·00	98·0	0·39	–0·15, 0·93	98·7	0·50	–0·11, 1·00
Fruit-flavoured drinks	220	54·1	0·0	0·0	45·9	0·00		45·9	0·00		–	–	
Nectars	160	2·5	0·0	0·0	97·5	0·00		97·5	0·00		–		
Coffee and tea	94	5·3	0·0	0·0	94·7	0·00		94·7	0·00		–		
Other beverages	285	35·8	0·4	0·7	64·6	0·01	–0·01, 0·04	64·9	0·03	–0·01, 0·06	99·7	0·67	0·05, 1·00
Dairy													
Sweetened dairy beverages	181	7·2	0·0	0·0	92·8	0·00		92·8	0·00		–	–	
Unsweetened dairy beverages	483	22·8	0·0	0·8	77·2	0·00		78·1	0·06	0·00, 0·11	99·2	0·00	
Cheese and cheese spreads	606	89·9	75·9	2·0	84·7	0·48	0·39, 0·56	12·1	0·01	0·00, 0·01	26·1	0·01	0·01, 0·02
Salty snacks	354	63·0	76·3	39·8	83·3	0·61	0·53, 0·70	62·2	0·28	0·19, 0·37	63·6	0·34	0·27, 0·41
Sweets and desserts													
Cookies	744	24·5	26·3	21·9	97·3	0·93	0·90, 0·96	93·2	0·81	0·76, 0·86	95·6	0·88	0·84, 0·92
Candies and desserts	1217	9·0	0·7	1·2	91·3	0·09	0·02, 0·16	91·6	0·16	0·07, 0·25	99·5	0·75	0·55, 0·94
Fruit preserves	407	0·2	0·2	0·0	99·5	0·00		99·8	0·00		99·8	0·00	
Convenience or ready-to-eat foods													
Frozen/fresh ready-to-eat foods	765	87·7	62·5	35·1	73·8	0·36	0·30, 0·41	47·5	0·14	0·11, 0·17	71·4	0·47	0·42, 0·52
Processed meats	807	93·8	83·5	50·7	89·0	0·47	0·38, 0·56	56·4	0·12	0·08, 0·15	67·2	0·34	0·29, 0·39
Sauces and dressings	798	80·2	69·8	50·4	87·6	0·68	0·62, 0·73	69·9	0·40	0·35, 0·45	80·1	0·60	0·55, 0·65
Bakery products	594	61·4	41·6	32·7	78·8	0·59	0·53, 0·65	69·9	0·44	0·38, 0·50	90·4	0·80	0·75, 0·85
Breakfast cereals and granola bars	308	3·9	3·6	3·2	98·4	0·77	0·58, 0·97	98·1	0·72	0·50, 0·93	99·7	0·95	0·85, 1·00
Culinary ingredients													
Sugar and non-nutritive sweeteners	104	1·0	0·0	1·0	99·0	0·00		100·0	1·00	1·00, 1·00	99·0	0·00	
Oils and fats	348	5·7	13·8	0·0	90·8	0·49	0·34, 0·64	94·3	0·00		86·2	0·00	
Other minimally processed and processed foods													
Canned vegetables	345	87·0	59·1	10·4	72·2	0·36	0·27, 0·44	23·5	0·03	0·02, 0·05	51·3	0·15	0·10, 0·20
Cereals, beans, other grain products	763	11·4	8·9	2·0	95·0	0·72	0·64, 0·81	90·6	0·28	0·17, 0·39	93·2	0·35	0·22, 0·47
Meat, poultry, seafood and egg	575	0·0	0·0	0·0									
Nuts and seeds	76	6·3	19·0	5·1	88·6	0·52	0·26, 0·78	89·9	0·15	0·20, 0·49	86·1	0·37	0·10, 0·64
Packaged fruits and vegetables	894	0·0	0·0	0·0									
Total	11 385	38·0	28·6	14·8	88·1	0·73	0·72, 0·75	75·8	0·42	0·40, 0·43	85·9	0·60	0·58, 0·61

PAHO, Pan American Health Organization; Anvisa, National Health Surveillance Agency (Agencia Nacional de Vigilância Sanitária); AG, agreement.

* It is not possible to calculate the kappa coefficient when there is no observation in one of the categories.

**Table 5:** Agreement between classifications made by assessed nutrient profiling models for high content of saturated fat applied to Brazilian packaged foods, 2017*

Food category	N	High content of saturated fat (%)			PAHO/Anvisa			PAHO/Chilean			Anvisa/Chilean		
		PAHO	Anvisa	Chilean	% AG	*κ*	95 % CI	% AG	*κ*	95 % CI	% AG	*κ*	95 % CI
Non-dairy beverages													
Carbonated beverages	106	0·0	0·0	0·0									
Fruit juices	150	0·0	0·0	0·0									
Fruit-flavoured drinks	220	0·0	0·0	0·0									
Nectars	160	0·0	0·0	0·0									
Coffee and tea	88	0·0	0·0	0·0									
Other beverages	284	9·9	7·0	0·0	97·2	0·82	0·70, 0·94	90·1	0·00		93·0	0·00	
Dairy													
Sweetened dairy beverages	181	8·8	16·0	0·0	80·7	0·12	–0·05, 0·29	91·2	0·00		84·0	0·00	
Unsweetened dairy beverages	483	60·9	5·8	5·6	44·9	0·08	0·05, 0·11	44·7	0·07	0·05, 0·10	99·0	0·90	0·82, 0·99
Cheese and cheese spreads	607	97·7	92·4	32·5	93·7	0·34	0·19, 0·50	34·8	0·02	0·01, 0·03	37·4	0·04	0·01, 0·06
Salty snacks	354	59·6	72·9	68·4	83·9	0·65	0·57, 0·73	85·6	0·69	0·61, 0·77	95·5	0·89	0·84, 0·94
Sweets and desserts													
Cookies	747	64·3	70·0	71·4	90·5	0·79	0·74, 0·83	91·6	0·81	0·76, 0·85	97·3	0·94	0·91, 0·96
Candies and desserts	1217	59·8	46·2	41·9	84·2	0·69	0·65, 0·73	80·8	0·63	0·59, 0·67	92·6	0·85	0·82, 0·88
Fruit preserves	409	10·3	10·8	2·2	95·6	0·77	0·66, 0·87	89·5	0·13	–0·01, 0·26	91·4	0·32	0·16, 0·47
Convenience or ready-to-eat foods													
Frozen/fresh ready-to-eat foods	794	50·1	27·5	23·8	74·8	0·50	0·44, 0·55	72·2	0·44	0·39, 0·50	96·4	0·90	0·87, 0·94
Processed meats	804	76·5	53·1	20·1	75·6	0·50	0·44, 0·55	43·7	0·14	0·12, 0·17	67·0	0·36	0·32, 0·41
Sauces and dressings	799	21·7	16·9	16·0	92·5	0·76	0·70, 0·82	91·6	0·73	0·67, 0·79	98·4	0·94	0·91, 0·97
Bakery products	594	22·6	22·4	22·4	95·5	0·87	0·82, 0·92	95·8	0·88	0·83, 0·93	99·7	0·99	0·98, 1·00
Breakfast cereals and granola bars	308	31·8	35·1	31·8	92·9	0·84	0·78, 0·90	92·9	0·84	0·77, 0·90	95·5	0·90	0·85, 0·95
Culinary ingredients													
Sugar and non-nutritive sweeteners	106	0·0	0·0	0·0									
Oils and fats	350	27·7	32·0	12·9	82·6	0·59	0·49, 0·68	85·1	0·56	0·46, 0·66	80·9	0·48	0·38, 0·57
Other minimally processed and processed foods													
Canned vegetables	343	22·7	7·3	3·5	83·4	0·38	0·26, 0·50	79·6	0·17	0·07, 0·27	96·2	0·63	0·45, 0·81
Cereals, beans, other grain products	727	2·1	5·6	1·0	95·1	0·34	0·18, 0·50	98·9	0·63	0·40, 0·87	95·3	0·28	0·12, 0·44
Meat, poultry, seafood and egg	573	0·0	0·0	0·0									
Nuts and seeds	79	13·9	73·4	17·7	40·5	0·11	0·04, 0·19	83·5	0·38	0·11, 0·65	44·3	0·15	0·06, 0·23
Packaged fruits and vegetables	848	0·0	0·0	0·0									
Total	11 331	35·4	29·0	20·4	87·7	0·72	0·71, 0·73	82·1	0·57	0·55, 0·58	90·5	0·75	0·73, 0·76

PAHO, Pan American Health Organization; Anvisa, National Health Surveillance Agency (Agência Nacional de Vigilância Sanitária); AG, agreement; CI, confidence interval.

* It is not possible to calculate the kappa coefficient when there is no observation in one of the categories.

Breakfast cereals and granola bars (71–72 %) as well as cookies (66–67 %) were some of the food categories with the largest proportion of products high in sugar, for which we found perfect to near-perfect agreement for all tested model comparisons (*κ* = 0·95–1·00). On the other hand, while 78·1 % of the sweetened dairy beverages were considered to have a high content of free sugars using the PAHO NPM, using the Anvisa NPM, only 3·1 % of the beverages in this category were high in free sugars. When we applied the Chilean NPM to the sample of sweetened dairy beverages, 40·6 % were found to be high in total sugars. For this group, not surprisingly we found only a slight agreement (*κ* = 0·02–0·09) when the NPM proposed by Anvisa was tested against both the PAHO (*κ* = 0·02) and the Chilean NPM (*κ* = 0·09) (Table 3). In Table 4, we present the prevalence of foods with high content of sodium and the agreement between the different NPM. Low to moderate agreement was found for frozen or fresh ready-to-eat foods, canned vegetables and processed meats (*κ* = 0·03–0·47). For instance, while 87·7 % of the frozen or fresh ready-to-eat foods were considered to be high in sodium using the PAHO NPM, 62·5 and 35·1 % of these foods were considered high in sodium when Anvisa and the Chilean NPM were applied. Similar differences were found for canned vegetables. The proportion of foods with high content of saturated fat in dairy beverages, cheeses and cheese spreads and processed meats had some of the lowest agreement when the different NPM were compared (Table 5).

**Fig. 1:**
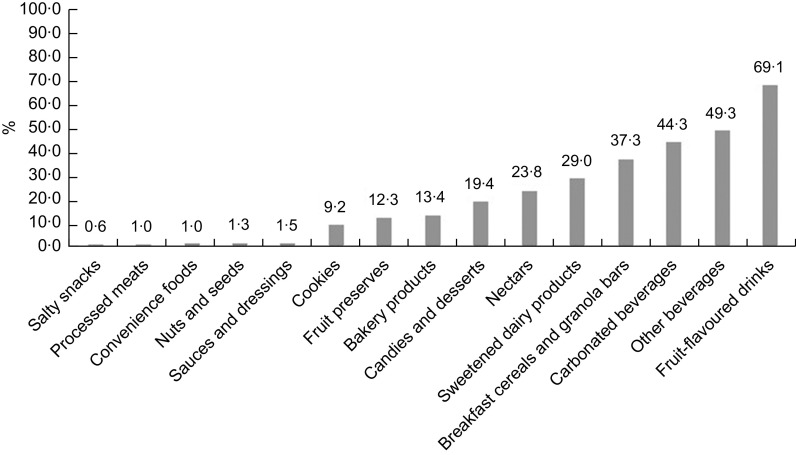
Presence of non-nutritive sweeteners in Brazilian packaged foods and beverages, 2017

We could not estimate the agreement between NPM for the presence of NNS because the Chilean and Anvisa proposed NPM do not consider their presence. We, however, depict the presence of NNS by food categories in Fig. 1. Overall, NNS were found in 10·0 % of the assessed Brazilian packaged foods. Non-dairy beverages such as fruit-flavoured drinks (69·1 %), carbonated beverages (44·3 %) and other non-dairy beverages (49·3 %) had the greatest proportion of foods/beverages with the presence of NNS. Also, NNS were found in over a third of breakfast cereals and granola bars and 29·0 % of sweetened dairy beverages.

The modified PAHO NPM model was also tested against the originally proposed PAHO model, and results are presented in online Supplementary file 3. PAHO’s and modified-PAHO’s NPM behaved similarly. When we considered only foods for which we were able to compute the amount of free sugars (10·9 % of the total sample), we found similar results.

## Discussion

In our study with a large sample of the Brazilian packaged foods from the five largest supermarket chains in Brazil, we found that the proportion and types of foods and beverages that would be classified as having a high content of critical nutrients and would be required to receive FOP warning labels vary depending on which NPM is chosen. Greatest disagreements were found for some of the food and beverages categories, particularly sweetened non-dairy and dairy beverages. The PAHO NPM identified a greatest proportion of sweetened non-dairy and dairy beverages with a high content of critical nutrients among the tested NPM. The same was found for ready-to-eat meals and processed meats for both sodium and saturated fat high content. The modified version of PAHO NPM behaved similarly to the originally proposed PAHO model and could be used for regulatory purposes when the eligibility criteria of the PAHO NPM cannot be used.

Consistent with our results, previous studies in Canada, Mexico, Australia, New Zealand and Malaysia have shown that the choice of which NPM to use has a large impact on what products are eligible to receive FOP nutritional labelling, have marketing restrictions, and/or be included in regulatory policies such as the restriction of sales of unhealthy foods in schools^(30–33)^. Our study adds to the literature by confirming the importance of carefully examining the characteristics of different NPM that could be adapted to be used in a specific food and nutrition policy.

In our study, we found Anvisa’s proposed NPM to be less strict for many of the food and beverage groups when we compared it with the PAHO NPM and the Chilean NPM, which, in turn, was less stringent than PAHO NPM for certain food groups. The greatest differences were found for beverages – both dairy and non-dairy beverages. PAHO NPM consider the products nutrient density (grams of sugar per kJ/kcal) as opposed to a volumetric measure (grams of sugar in 100 ml/g of a product) used on the Anvisa and the Chilean NPM. Major disagreements were found for low-energy beverages. For example, 100 ml of a reduced sugary beverage sold in Brazil has 4·5 g of sugar and 75 kJ (18 kcal). This amount of sugar is below the 10 % daily limit adopted in Anvisa NPM, and the product would not carry FOP warning labels with this model. On the other hand, the PAHO NPM considers that all the energy content of this product is derived from free sugar, and for that reason FOP warning labels would be presented. Considering an average serving (350 ml) of such low-energy beverage provides 15·7 g of added sugars (31·5 % of the recommended daily limit of sugar), Anvisa NPM cut-off points for sugars may not be adequate for alerting consumers which products have high levels of added sugars. Despite being able to identify more sweetened beverages than the model proposed by Anvisa, the Chilean NPM identified a smaller number of sweetened beverages than the PAHO NPM. Considering sweetened beverages contribute with 49 % of the added sugars consumption in Brazil^(34)^, misclassifying such foods could jeopardise the ability of the FOP to help consumers make healthier choices that can result in lower consumption of added sugars. The inability to correctly classify sweetened dairy beverages as high in sugars could particularly impact the consumption of added sugars among Brazilian children and adolescents. A third of Brazilian children under the age of 2 years consume sweetened beverages^(35)^. Adolescents report sweetened dairy beverages among the most consumed food and^(36)^ have a higher proportional contribution of sweetened dairy beverages to added sugars intake than adults and elders^(34)^.

In addition, our study is the first to test which foods would be required to receive FOP warning labels for the presence of NNS. While Anvisa’s proposed NPM and the NPM used in Chile do not consider the presence of NNS in foods and beverages, PAHO NPM do. Some of the beverages that would not be required to carry FOP warning labels for high content of sugars, would, however, be required to carry FOP warning labels for the presence of NNS should PAHO NPM be implemented. Our findings in Brazil corroborate similar studies conducted in other Latin American countries and previously for a smaller sample of packaged foods in Brazil^(37)^. In Mexico, 11 % of foods and beverages – for which information was collected in large supermarket chains using similar methods as those applied in our study – had NNS^(38)^. NNS were found in a smaller percentage of foods and beverages in countries in North America and Oceania^(38)^. On the supply side, fiscal policies aimed at increasing the price of sugar-sweetened beverages are also likely to push for the reformulation of sugary beverages involving partial or total substitution of added sugars for NNS. Although the evidence that links the consumption of NNS to health outcomes is not conclusive, recent studies have found that their intake is associated with weight gain^(39)^ and increased risk of stroke, CHD and all-cause mortality^(40)^. In addition, most of the NNS categories already have a maximum limit of use established by the Brazilian government based on the potential risks^(24)^. Taken together, better informing consumers about the presence of NNS in foods and beverages while more studies that link the consumption of NNS with health outcomes are conducted could prevent potential future harm, particularly among children for whom evidence on the long-term effects of NNS use is more limited^(41)^. Toddlers are an even more concerning group considering NNS should not be part of their diet^(42)^. Because of the increasing use of NNS in foods and beverages, further evidence on their contemporary intake, including type and amount, is warranted; as well as more information about the type and quantity of NNS present in various foods and beverages^(41)^. Including clearer information for the presence of NNS in foods and beverages could also nudge the food industry to lower the content of added sugars in products with a high content of total or added sugars without the obvious substitution for NNS^(43)^.

In addition to cut-off points and which nutrients to consider, the eligibility criteria or which foods and beverages are eligible to be regulated is a matter just as important when classifying foods for regulatory purposes. As opposed to the Anvisa NPM, PAHO NPM (processed and ultra-processed foods) and the Chilean NPM (foods and beverages with added critical nutrients – sugar, sodium and saturated fat – that have been linked to non-communicable diseases) have criteria to objectively determine which foods are eligible to be regulated and therefore receive FOP warning labels. Moreover the Anvisa NPM excludes foods for special needs, including sugar-free foods and beverages and products for toddlers.

Another important issue when determining the most adequate NPM for each context includes the implications of having too many foods with FOP warning labels in the marketplace. Considering FOP warning labels have only more recently been implemented^(7)^, no evidence is yet available on the effects of a very strict NPM. When a NPM is so strict that a large proportion of food items from a specific category have warnings, it may lead to no stimulus for the consumer to choose the healthiest option within that specific category – nor for the food industry to reformulate. However, having most food items with FOP warning labels in one single category where most if not all foods are a known source of critical nutrients, such as sugar-sweetened beverages, could nudge consumers to switch between categories and ultimately improve their diet. Experiments and evaluations of labelling policies recently implement across the globe using different NPM, and cut-offs are needed to help policy makers define an optimum NPM.

Our study has a few limitations. First, we did not weigh the products by market share and did not specifically consider the most consumed foods. However, we included a considerable sample (over 10 000 items) of foods sold by the five top grocery retailers in Brazil. The selected retailers account for almost 70 % of the grocery retail market share in the country^(19)^. And foods purchased at supermarkets account for 59 % of the energies consumed in Brazil^(17)^. We estimate that if we were able to weigh our food categories by consumption share, we would have found an even greater disagreement between the PAHO NPM and the Anvisa or Chilean NPM in sweetened dairy and non-dairy beverages, considering their high consumption in the Brazilian population, particularly among adolescents^(34,36,44)^. Second, we only included packaged foods that have information on the ingredients list and a nutrition facts panel. Third, although several NPM exist worldwide, we decided to include only those that have been relevant in the debate for nutritional criteria in Brazil and Latin America, which strengthens evidence-based regulatory processes^(16)^. Applying a NPM that uses nutrient thresholds that have been linked to the prevention of diet-related diseases in the design of regulatory food policies can improve the effectiveness of the policy to reach its goals to help curb growing diet-related disease rates. Moreover, having harmonised systems across Latin America would be valuable for consumers and manufacturers.

In conclusion, we found variations in the degree of strictness and agreement between NPM, particularly among beverages. The PAHO NPM identified more sweetened beverages high in sugar and those with the presence of NNS. The same was found for certain foods high in sodium and saturated fat. Our results highlight the importance of carefully evaluating the characteristics of NPM for use in food and public health policies, such as nutritional labelling, so such policies achieve their goals in helping consumers select healthier foods. Considering the growing epidemiological and economic burden of obesity and diabetes in Brazil^(45,46)^, evidence-based criteria and regulations aligned with international recommendations can help policy makers design more effective measures to help consumers make healthier choices and to promote reformulation by the food industry.

## Supplementary material

For supplementary material accompanying this paper visit https://doi.org/10.1017/S1368980019005056. click here to view supplementary material
