# Supplementary material for: Role of different nutrient profiling models in identifying targeted foods for front-of-package food labelling in Brazil
Source: Public Health Nutr. 2020 Jun 9;24(6):1514–25. doi: 10.1017/S1368980019005056 (PMC8025091; doi:10.1017/S1368980019005056)
Supplement: Supplementary file 1 [file S1368980019005056sup.zip › S1368980019005056sup001.docx]

| **Supplementary file 2.** Description of the food categories. | |
| --- | --- |
| **Food category** | **Foods included** |
| Non-dairy beverages | |
| Carbonated beverages | Carbonated beverages, including artificially sweetened versions. |
| Fruit juices | Products declared as juices, fruit juices without water or added sugar, coconut water. |
| Fruit-flavored drinks | Fruit drink powder, fruit punch concentrate, fruit-flavored beverages. |
| Nectars | Nectars made with juice and added water and/or sugar. |
| Coffee and tea | Coffee grains and powder, tea sachets and leaves. |
| Other beverages | Plant-based beverages, ready-to-drink teas, isotonic drinks, coconut milk. |
| Dairy | |
| Sweetened dairy beverages | Sweetened yogurt, flavored milks, fermented milk, milk compounds, dairy beverages. |
| Unsweetened dairy beverages | Unsweetened yogurt, milks, evaporated and powdered milk, milk compounds. |
| Cheese and cheese spreads | Processed and ultra-processed cheese, cheese spreads. |
| Salty snacks | Salted peanuts, salty snacks, potato chips, potato sticks, microwave popcorn. |
| Cookies and crackers | Sweet and savory crackers, biscuits, and cookies. |
| Sweets, desserts, and convenience foods | |
| Candies and desserts | Chocolate bars, candies, condensed milk, cocoa powder and sweetened dairy mixes, jellies, syrups, chocolate spreads, chewing gum, marshmallows, ice cream (including versions with nonnutritive sweeteners). |
| Fruit preserve | Fruit jellies, fruit preserves, canned fruits, dried fruits, fruit sorbets. |
| Convenience foods | Ready-to-eat meals, frozen french fries, instant rice, instant noodles, instant soups, instant mashed potatoes, stuffed pasta, frozen pizzas, frozen and ready-to-eat pies, sandwiches, baby foods. |
| Processed meats | Burgers, sausages, canned fish, smoked meats, seasoned meats, salted meats, hams, salami, spreads |
| Sauces, herbs, and dressings | Sauces, mayonnaises, herbs, catchup, salad dressings. |
| Bakery products | Breads, toasts, and cakes (including powders). |
| Breakfast cereals and granola bars | Corn flakes, flavored oats, infant cereals, granolas, mueslis, granola bars, porridges, mix of cereals and fruits. |
| Culinary ingredients | |
| Sugar and other nonnutritive sweeteners | Sugar, honey, nonnutritive sweeteners. |
| Oils and fats | Oils, margarines, butters, fresh cream, fats. |
| Other minimally processed and processed foods | |
| Canned vegetables | Canned beans and vegetables. |
| Cereals, beans, other grain products | Dry beans, flours, rice, corn, pasta. |
| Meat, poultry, seafood, and egg | Meat, poultry, seafood, egg, including chilled and frozen products. |
| Nuts and seeds | Nuts and seeds, including salted nuts. |
| Packaged fruits and vegetables | Fresh and frozen fruits and vegetables, frozen fruit pulp. |
