# Supplementary material for: Role of different nutrient profiling models in identifying targeted foods for front-of-package food labelling in Brazil
Source: Public Health Nutr. 2020 Jun 9;24(6):1514–25. doi: 10.1017/S1368980019005056 (PMC8025091; doi:10.1017/S1368980019005056)
Supplement: Supplementary file 1 [file S1368980019005056sup.zip › S1368980019005056sup002.docx]

* Only single ingredient products without the list of ingredients were kept in the database.

12,956 items

**Exclusions for the current analysis:**

Products available in more than one package size (n=358)

Multipack with different items (n=86)

Products without nutrition facts panel (n=815)

Products without the list of ingredients* (n=178)

Products with missing values for portion size and/or calories (n=85)

14,877 items

**General exclusions:**

Training records (n= 405)

Items photographed more than once (n=1,514)

Supplements (n=2)

11,434 items

Supplementary file 1. Flow chart of exclusions in the study.
