# Supplementary material for: Role of different nutrient profiling models in identifying targeted foods for front-of-package food labelling in Brazil
Source: Public Health Nutr. 2020 Jun 9;24(6):1514–25. doi: 10.1017/S1368980019005056 (PMC8025091; doi:10.1017/S1368980019005056)
Supplement: Supplementary file 1 [file S1368980019005056sup.zip › S1368980019005056sup003.docx]

| Supplementary file 3. Agreement between the modified Pan American Health Organization nutrient profiling model and the original version for high content of critical nutrients applied to Brazilian packaged foods*, 2017. | | | | | | | | | | | | | | |
| --- | --- | --- | --- | --- | --- | --- | --- | --- | --- | --- | --- | --- | --- | --- |
| Food category | Full sample | | Products high in critical nutrients (%) | | | High content of sugars* (%) | | | High content of sodium (%) | | | High content of saturated fat (%) | | |
|  | n | % | % | 95%CI | | % of products | % AG. | k (95%CI) | % of products | % AG. | k (95%CI) | % of products | % AG. | k (95%CI) |
| Non-dairy beverages |  |  |  |  |  |  |  |  |  |  |  |  |  |  |
| Carbonated beverages | 106 | 0.9 | 57.5 | 47.9 | 66.6 | 58.8 | 100.0 | 1.000 (1.000 - 1.000) | 1.0 | 76.2 | 0.057 (-0.050 - 0.164) | 0.0 | . | . |
| Fruit juices | 150 | 1.3 | 12.0 | 7.7 | 18.3 | 25.4 | 89.8 | 0.691 (0.468 - 0.914) | 2.0 | 99.3 | 0.854 (0.571 - 1.000) | 0.0 | . | . |
| Fruit-flavored drinks | 220 | 1.9 | 73.6 | 67.4 | 79.0 | 62.7 | 100.0 | 1.000 (1.000 - 1.000) | 0.0 | 45.9 | 0.000 | 0.0 | . | . |
| Nectars | 160 | 1.4 | 28.8 | 22.3 | 36.3 | 77.3 | 100.0 | 1.000 (1.000 - 1.000) | 0.0 | 97.5 | 0.000 | 0.0 | . | . |
| Coffee and tea | 94 | 0.8 | 0.0 | . | . | 0.0 | . | . | 0.0 | 94.7 | 0.000 | 0.0 | . | . |
| Other beverages | 286 | 2.5 | 65.4 | 59.7 | 70.7 | 52.1 | 98.6 | 0.971 (0.932 - 1.000) | 13.0 | 77.2 | 0.422 (0.322 - 0.523) | 2.1 | 92.3 | 0.330 (0.130 - 0.529) |
| Dairy |  |  |  |  |  |  |  |  |  |  |  |  |  |  |
| Sweetened dairy beverages | 483 | 4.2 | 50.7 | 46.3 | 55.2 | 78.1 | 100.0 | 1.000 (1.000 - 1.000) | 0.0 | 92.8 | 0.000 | 0.6 | 91.7 | 0.108 (-0.087 - 0.304) |
| Unsweetened dairy beverages | 181 | 1.6 | 0.6 | 0.1 | 3.8 | 0.0 | . | . | 2.9 | 80.1 | 0.184 (0.099 - 0.268) | 17.6 | 56.7 | 0.241 (0.191 - 0.292) |
| Cheese and cheese spreads | 607 | 5.3 | 90.9 | 88.4 | 93.0 | 0.0 | . | . | 88.9 | 98.7 | 0.930 (0.882 - 0.978) | 34.3 | 36.6 | 0.024 (0.011 - 0.037) |
| Salty snacks | 356 | 3.1 | 91.9 | 88.5 | 94.3 | 1.8 | 100.0 | 1.000 (1.000 - 1.000) | 61.6 | 98.6 | 0.970 (0.944 - 0.996) | 58.2 | 98.6 | 0.971 (0.945 - 0.996) |
| Sweets and desserts |  |  |  |  |  |  |  |  |  |  |  |  |  |  |
| Cookies | 747 | 6.5 | 93.0 | 91.0 | 94.7 | 65.9 | 100.0 | 1.000 (1.000 - 1.000) | 24.1 | 99.6 | 0.989 (0.977 - 1.000) | 63.7 | 99.5 | 0.988 (0.977 - 1.000) |
| Candies and desserts | 1220 | 10.7 | 71.8 | 69.2 | 74.3 | 70.6 | 100.0 | 1.000 (1.000 - 1.000) | 6.5 | 97.5 | 0.827 (0.767 - 0.888) | 51.0 | 91.2 | 0.823 (0.792 - 0.855) |
| Fruit preserves | 414 | 3.6 | 16.7 | 13.4 | 20.6 | 45.2 | 100.0 | 1.000 (1.000 - 1.000) | 0.0 | 99.8 | 0.000 | 2.4 | 89.7 | 0.159 (0.019 - 0.299) |
| Convenience or ready-to-cook foods |  |  |  |  |  |  |  |  |  |  |  |  |  |  |
| Frozen and fresh ready-to-cook foods | 795 | 7 | 90.9 | 88.7 | 92.8 | 3.3 | 100.0 | 1.000 (1.000 - 1.000) | 86.1 | 97.7 | 0.900 (0.855 - 0.946) | 44.6 | 94.5 | 0.889 (0.858 - 0.921) |
| Processed meats | 810 | 7.1 | 94.2 | 92.4 | 95.6 | 0.0 | . | . | 91.6 | 97.8 | 0.836 (0.762 - 0.910) | 32.5 | 56.0 | 0.257 (0.219 - 0.296) |
| Sauces and dressings | 801 | 7 | 82.3 | 79.5 | 84.8 | 47.4 | 100.0 | 1.000 (1.000 - 1.000) | 78.9 | 97.5 | 0.923 (0.890 - 0.956) | 21.4 | 98.8 | 0.963 (0.940 - 0.986) |
| Bakery products | 595 | 5.2 | 83.7 | 80.5 | 86.5 | 18.2 | 100.0 | 1.000 (1.000 - 1.000) | 58.9 | 97.5 | 0.947 (0.921 - 0.974) | 22.6 | 100.0 | 1.000 (1.000 - 1.000) |
| Breakfast cereals and granola bars | 308 | 2.7 | 62.0 | 56.5 | 67.3 | 71.1 | 100.0 | 1.000 (1.000 - 1.000) | 3.9 | 100.0 | 1.000 (1.000 - 1.000) | 28.2 | 96.4 | 0.915 (0.866 - 0.964) |
| Culinary ingredients |  |  |  |  |  |  |  |  |  |  |  |  |  |  |
| Sugar and nonnutritive sweeteners | 106 | 0.9 | 42.5 | 33.4 | 52.1 | 0.0 | . | . | 0.0 | 99.0 | 0.0 | 0.0 | . | . |
| Oils and fats | 351 | 3.1 | 16.8 | 13.2 | 21.1 | 0.0 | . | . | 8.3 | 97.4 | 0.803 (0.678 - 0.928) | 12.9 | 85.1 | 0.556 (0.456 - 0.656) |
| Other minimally processed and processed foods |  |  |  |  |  |  |  |  |  |  |  |  |  |  |
| Canned vegetables | 345 | 3 | 88.4 | 84.6 | 91.4 | 0.0 | . | . | 86.4 | 99.4 | 0.975 (0.940 - 1.000) | 7.6 | 84.8 | 0.436 (0.319 - 0.552) |
| Cereals, beans, other grain products | 735 | 6.4 | 11.7 | 9.6 | 14.2 | 0.0 | . | . | 11.0 | 99.6 | 0.980 (0.956 - 1.000) | 1.5 | 99.5 | 0.843 (0.692 - 0.995) |
| Meat, poultry, seafood, and egg | 577 | 5 | 0.0 | . | . | 0.0 | . | . | 0.0 | . | . | 0.0 | . | . |
| Nuts and seeds | 80 | 0.7 | 28.8 | 19.9 | 39.6 | 0.0 | . | . | 6.3 | 98.7 | 0.902 (0.713 - 1.000) | 7.6 | 93.7 | 0.674 (0.412 - 0.935) |
| Packaged fruits and vegetables | 907 | 7.9 | 0.0 | . | . | 0.0 | . | . | 0.0 | . | . | 0.0 | . | . |
| Total | 11,434 | 100 | 57.6 | 56.7 | 58.5 | 52.4 | 99.4 | 0.987 (0.978 - 0.996) | 34.2 | 95.9 | 0.912 (0.904 - 0.920) | 23.9 | 88.3 | 0.725 (0.711 - 0.738) |
| Note: It is not possible to calculate the kappa coefficient when there is no observation in one of the categories. | | | | | | | | | | | | | | |
| Abbreviations: CI, confidence interval; PAHO, Pan American Health Organization; Anvisa, National Health Surveillance Agency (Agência Nacional de Vigilância Sanitária); AG=Agreement | | | | | | | | | | | | | | |
| *Information for the content of free or total sugars were considered in 10% of the sample. For the PAHO and Anvisa NPM, free sugars were calculated from the information on total sugars available on products (n=1,242). For the Chilean NPM, the information on total sugars content available on food products nutrition facts panels was considered (n=1,242). | | | | | | | | | | | | | | |
|  |  |  |  |  |  |  |  |  |  |  |  |  |  |  |
